# Immunogenicity and Protective Efficacy of T-Cell Epitopes Derived From Potential Th1 Stimulatory Proteins of *Leishmania (Leishmania) donovani*

**DOI:** 10.3389/fimmu.2019.00288

**Published:** 2019-02-28

**Authors:** Sumit Joshi, Narendra Kumar Yadav, Keerti Rawat, Vikash Kumar, Rafat Ali, Amogh Anant Sahasrabuddhe, Mohammad Imran Siddiqi, Wahajul Haq, Shyam Sundar, Anuradha Dube

**Affiliations:** ^1^Parasitology Division, Council of Scientific & Industrial Research-Central Drug Research Institute, Lucknow, India; ^2^Molecular and Structural Biology Division Council of Scientific & Industrial Research-Central Drug Research Institute, Lucknow, India; ^3^Medicinal Process Chemistry Division, Council of Scientific & Industrial Research-Central Drug Research Institute, Lucknow, India; ^4^Department of Medicine, Institute of Medical Sciences, Banaras Hindu University, Varanasi, India

**Keywords:** visceral leishmaniasis, Th1 stimulatory proteins, immunoinformatics, T-cell epitopes, peptides, human PBMCs, hamsters, protective response

## Abstract

Development of a suitable vaccine against visceral leishmaniasis (VL), a fatal parasitic disease, is considered to be vital for maintaining the success of kala-azar control programs. The fact that *Leishmania*-infected individuals generate life-long immunity offers a viable proposition in this direction. Our prior studies demonstrated that T-helper1 (Th1) type of cellular response was generated by six potential recombinant proteins *viz*. elongation factor-2 (elF-2), enolase, aldolase, triose phosphate isomerase (TPI), protein disulfide isomerase (PDI) and p45, derived from a soluble antigenic fraction (89.9–97.1 kDa) of *Leishmania (Leishmania) donovani* promastigote, in treated *Leishmania* patients and golden hamsters and showed significant prophylactic potential against experimental VL. Moreover, since, it is well-known that our immune system, in general, triggers production of specific protective immunity in response to a small number of amino acids (peptide), this led to the identification of antigenic epitopes of the above-stated proteins utilizing immunoinformatics. Out of thirty-six, three peptides-P-10 (enolase), P-14, and P-15 (TPI) elicited common significant lymphoproliferative as well as Th1-biased cytokine responses both in golden hamsters and human subjects. Further, immunization with these peptides plus BCG offered 75% prophylactic efficacy with boosted cellular immune response in golden hamsters against *Leishmania* challenge which is indicative of their candidature as potential vaccine candidates.

## Introduction

Visceral leishmaniasis (VL or kala-azar), caused by *Leishmania (Leishmania) donovani* and *Leishmania (Leishmania) infantum* is the most severe form of leishmaniasis wherein the immune system of the affected individuals is severely impaired making them susceptible to secondary infections (1). Brazil, Ethiopia, India, Kenya, Somalia, South Sudan, and Sudan accounts for more than 90% of the recent VL cases (2). Though there is a drastic reduction in a number of active VL patients due to the available chemotherapeutics, the presence of potential reservoirs (asymptomatic and post kala-azar dermal leishmanoids patients) enforce the need of an effective VL vaccine in order to persist the success of current VL elimination program (3). The fact, that there is a development of protective immunity in VL patients in endemic areas, either naturally or after treatment, indicates toward the feasibility of a VL vaccine (4). Although canine VL vaccines are commercially available, no licensed vaccine has been developed against human VL till date (5).

Nowadays, vaccine designing becomes more refined with the improvement in the knowledge of targets of immune responses (6). Peptide epitope, the minimal immunogenic regions of a protein antigen, are the most precise vaccine components that guide the direction of immune responses, hence, forms the basis of peptide-based synthetic vaccines (7). Generation of optimal immune response with these vaccines requires better interaction between T- and B- cell epitopes of pathogen proteins and major histocompatibility complex (MHC) molecule of the host (8). Recent immunoinformatics approaches exploit various algorithms which can predict epitopes with the highest probability of inducing protective immune responses (9). A number of such vaccines have been evaluated against various parasitic diseases such as malaria, toxoplasmosis, and trypanosomiasis (10–13). These vaccines offer several merits over conventional ones particularly in terms of safety and regulatory issues and ease of production (7).

Development of Th1 type immune response, produced by CD4+ T cells, plays a vital role in mediating protection against intracellular pathogens including *Leishmania* (14), hence, T-cell stimulatory antigens have been explored as suitable vaccine candidates (15). Previously, in our laboratory, proteomic analysis of F2 fraction (ranging from 89.9 to 97.1 kDa) of soluble *L*. (*L*.) *donovani* (SLD) antigen revealed eighteen Th1 stimulatory proteins (16) out of which fifteen were developed as recombinant ones. Comparative immunogenicity of these fifteen recombinant proteins helped in the identification of six *viz*. aldolase, elongation factor-2 (elF-2), enolase, protein disulfide isomerase (PDI), p45 and triose phosphate isomerase (TPI) that elicited an optimum cellular response (17). In this study, we further undertook identification of potential T-cell epitopes from the above-stated six potential recombinant Th1 stimulatory proteins using immunoinformatics and evaluated the cellular responses in lymphocytes/PBMCs of treated *Leishmania*-infected hamsters/patients as well as the prophylactic efficacies of these peptides in golden hamster against *L. (L.) donovani* challenge. These peptides if found potential may further lead to the development of a chimeric vaccine for a specific and optimum immunity against VL.

## Materials and Methods

### Expression and Purification of Recombinant Proteins

Six recombinant Th1 stimulatory proteins namely aldolase, enolase, elF-2, p45, PDI, and TPI (Accession numbers: GQ220750.1, EU723850.1, EU929069.1, EU723851.1, EU723849.1, and EU867389.1, respectively), were expressed and purified using previously optimized protocols (18–22) and were used herein to compare the results obtained with the peptides identified in this study. The LPS content of the purified recombinant proteins was measured by *Limulus* amoebocyte lysate test (Pierce LAL Chromogenic Endotoxin Quantitation Kit, Thermo Scientific) and was found to be below 10 endotoxin units (EU)/mg of the recombinant protein.

### *In silico* Prediction and Chemical Synthesis of Peptides

MHC class II binding epitopes were predicted from the above-stated six Th1 stimulatory proteins using Immuno Epitope Database (IEDB) and SYFPEITHI and were synthesized at 0.1 mmol scales using Fmoc-Chemistry by standard solid phase peptide synthesis chemistry.

### Animal and Infection

Syrian golden hamsters served as a suitable animal model and were infected by amastigotes purified using percoll (Sigma-Aldrich, USA) density gradient centrifugation method, described by Chang (23). Briefly, the spleens from *Leishmania*-infected hamsters (45–60 day old) were removed aseptically and homogenized in sterile phosphate buffer saline (PBS) at 4°C. The suspension was centrifuged at 900Xg for 10 min at 4°C, to settle the tissue debris and the supernatant was again centrifuged at 3000Xg for 10 min at 4°C. The sediment containing amastigotes, after RBC lysis treatment, was resuspended in 45% percoll solution in PBS-EDTA (2 mM) which was overlaid in 95% percoll solution and centrifuged at 3,500Xg for 1 h at 4°C. A middle creamy or yellowish layer of amastigotes was aspirated, washed twice and then finally suspended in PBS for counting using an automated cell counter. These purified amastigotes were further used for infecting hamsters.

A batch of 30 hamsters was used for *in vitro* study. 0.1 ml of inoculum per animal containing 2 × 10^6^ amastigotes/ml was inoculated in 20 hamsters intracardially (i.c.) whereas 10 hamsters were kept as uninfected control. One month later, the magnitude of infection in infected animals was assessed by rk39 dipstick (InBios International Inc., USA). Miltefosine (Synphabase, Switzerland), an oral anti-leishmanial drug, was administered in 10 out of 20 infected hamsters @ 40 mg/kg bodyweight daily for 5 days and these animals were referred as treated *Leishmania-*infected hamsters.

### *In vitro* Evaluation of Peptides

*In vitro* cellular proliferation assay was performed both in mononuclear cells isolated from mesenteric lymph nodes of infected, treated and uninfected hamsters, as well as in peripheral blood mononuclear cells (PBMCs) from human subjects (Supplementary Table I) using the Ficoll Hypaque density gradient centrifugation (Histopaque 1077, Sigma-Aldrich, USA) as described by Garg et al. (24). Briefly, 0.1 ml of cell suspension in complete RPMI-1640 (Sigma-Aldrich, USA) containing 1 × 10^6^ cells/ml was seeded in 96-well flat-bottom tissue culture plates (Nunc, Denmark) and stimulated with each of the 6 recombinant proteinsand their total 36 peptides @ 10 ng/ml in triplicates. Phytohaemagglutinin (PHA, 10 ng/ml Sigma-Aldrich, USA) for Patient's PBMCs and concanavalin A (ConA, 10 ng/ml, Sigma-Aldrich, USA) for hamster's mononuclear cells were used as standard mitogens to ascertain the procedural sensitivity. Cells were maintained in a CO_2_ incubator (37°C with 5% CO_2_) for 3 days. After the incubation, 100 μl of supernatant was taken out from each well and 50 μl of XTT (Biological Industries, Israel) was added to the remaining media containing cells for 4 h before the termination of the experiment. The absorbance of the samples, at 480 nm with 650 nm as a reference wavelength, was measured in a SPECTRAmax PLUS 384 microplate reader (Molecular Devices, USA). Wells without stimulants served as blank and their values were subtracted from the corresponding stimulated one.

Cytokine production was also estimated in cell culture supernatants of 3 potential peptide-stimulated cells by ELISA [(MyBioSource, USA (for hamster) and BD Pharmingen, USA (for human)] according to the manufacturer's protocol. The amount of cytokine production noticed in cell culture supernatants from unexposed healthy hamsters and humans was deducted from the infected one.

### *In vivo* Evaluation of Peptides

A total of 60 hamsters was divided into 6 groups (*n* = 10/group) for efficacy study of 3 potential peptides based on the results of *in vitro* study. Of the six groups, hamsters belonging to group I to III were immunized intradermally (i.d.) on the footpad with each of the three peptides (P-10, P-14, and P-15) at a dose of 50 μg/50 μl/animal emulsified with an equal volume of BCG, whereas group IV received BCG alone. Fourteen days later, a booster with half of the first dose of the respective peptide plus BCG was given by intradermal route (i.d.) to all hamsters of Group I to III and Group IV received BCG alone. Hamsters of groups I to V were challenged intracardially (i.c.) 14 days after the last booster dose with 2 × 10^5^ *L. (L.) donovani* purified amastigotes per animal. The amastigotes were isolated from the spleen of heavily infected (50–60 days old) hamsters as described previously (section Animal and infection). Hamsters in Group V and VI were kept as infected and uninfected controls, respectively. Five hamsters from each group were sacrificed on days 60 and 90 post-challenge (p.c.) for the assessment of parasite load and other immunological parameters. The parasite load was assessed as the number of amastigotes/100 cell nuclei in the Giemsa stained splenic dab-smears and expressed in percentage inhibition (PI) using the following formula (18).

$$P1=\frac{\left(\begin{array}{l} \text{No}.\text{ of parasite from infected control}-\\ \text{ No}.\text{ of parasite from vaccinated group} \end{array}\right)}{\text{No}.\text{ of parasite count from infected control}}\times 100$$

Delayed-type hypersensitivity (DTH) assay was performed on days 60 and 90 p.c. as per the protocol described by Kumari et al. (25) on the contralateral footpad and the response was measured 24 h later in all the experimental groups. Evaluation of lymphoproliferative response was carried out in mesenteric lymph node-derived mononuclear cells from all the experimental groups on days 60 and 90 p.c. as per method described previously (24). The levels of Th1 and Th2 cytokines were measured in the hamster's serum by ELISA (MyBioSource, USA). In addition, the transcript of these cytokines were also estimated in their splenic tissues through quantitative Real-time RT-PCR (iQ5 LightCycler, BioRad) using the following reaction conditions: initial denaturation at 95°C for 2 min followed by 40 cycles, each consisting of denaturation at 95°C for 20 s, annealing at 60°C for 20 s and extension at 72°C for 16 s per cycle employing various sets of primers (Table 1). The mRNA expression of target genes was normalized to RPL18 (housekeeping gene) using the 2^−ΔΔCt^ method (26) wherein the PCR signal of the target transcript in a treatment group is related to that of untreated control.

**Table 1:** Sequences of forward and reverse primers of hamster cytokines used for quantitative real time RT-PCR.

**S. No**.	**Genes**	**Direction**	**Primer sequence**	**Product length**
1	RPL 18	Forward Reverse	5′ GATAGATCCACTCCCATAACTG 3′ 5′ TACCTTCAACAATCAAGACATTC 3′	96
2	TNF-α	Forward Reverse	5′ TTCTCCTTCCTGCTTGTG 3′ 5′ CTGAGTGTGAGTGTCTGG 3′	131
3	IFN-γ	Forward Reverse	5′ GCTTAGATGTCGTGAATGG 3′ 5′ GCTGCTGTTGAAGAAGTTAG 3′	200
4	IL-12	Forward Reverse	5′ TATGTTGTAGAGGTGGACTG 3′ 5′ TTGTGGCAGGTGTATTGG 3′	81
5	TGF-β	Forward Reverse	5′ ACGGAGAAGAACTGCTGTG 3′ 5′ GGTTGTGTTGGTTGTAGAGG 3′	178
6	IL-4	Forward Reverse	5′ GCCATCCTGCTCTGCCTTC 3′ 5′ TCCGTGGAGTTCTTCCTTGC 3′	75
7	IL-10	Forward Reverse	5′ TGCCAAACCTTATCAGAAATG 3′ 5′ AGTTATCCTTCACCTGTTCC 3′	102

### Statistical Analysis

**Figure 1:**
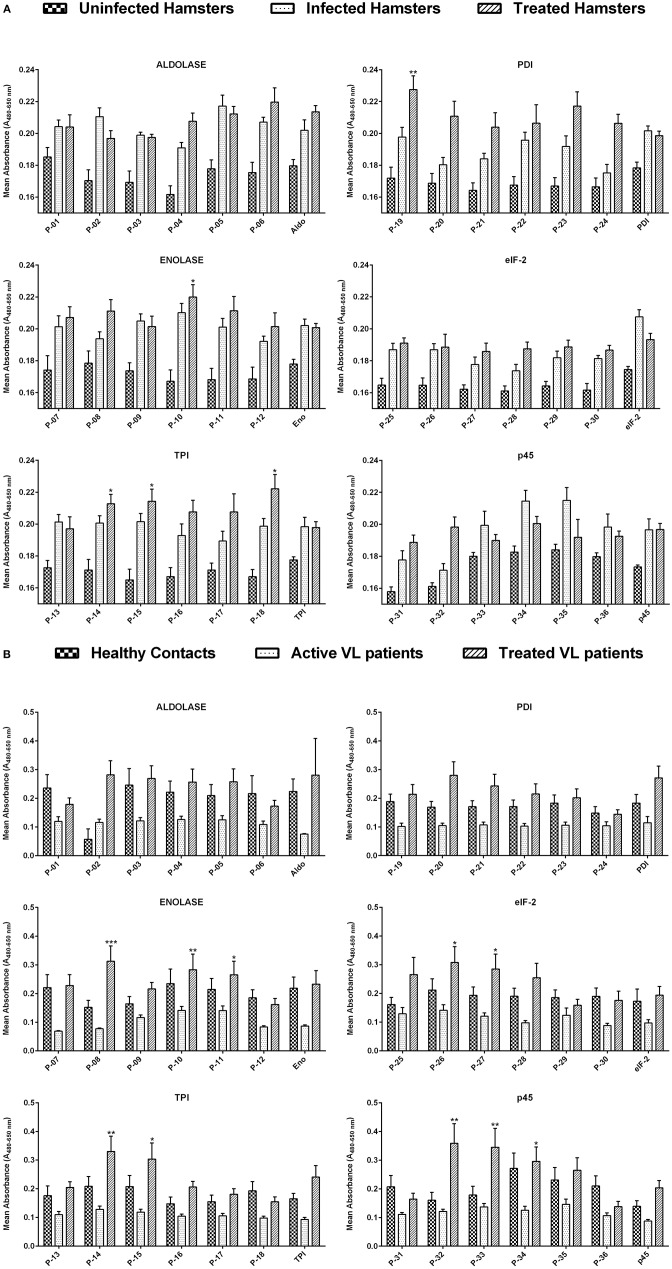
**(A)** Lymphoproliferative response in lymphocytes derived from the mesenteric lymph node of uninfected, infected as well as treated *Leishmania*-infected hamsters against 36 Th1 stimulatory peptides in comparison to their parent recombinant proteins. The concentration of peptides, as well as recombinant proteins, was adjusted to 1 ng/ml of cells. Each bar represents the pooled data of hamsters (Mean ± SE). Statistical significant differences were assessed using a paired *t*-test amid mean values of treated groups stimulated with synthetic peptides and their respective recombinant proteins. **(B)** Lymphoproliferative response in PBMCs of healthy contacts, active VL patients as well as treated VL patients against 36 Th1 stimulatory peptides and their parent recombinant proteins. The concentration of peptides, as well as recombinant proteins, was adjusted to 1 ng/ml of cells. Each bar represents the pooled data (Mean ± SE) and the levels of statistical significance were assessed between the mean values of treated groups, stimulated with synthetic peptides and their respective recombinant proteins by paired *t*-test. *p < 0.05; **p < 0.01; and ***p < 0.001.

**Figure 2:**
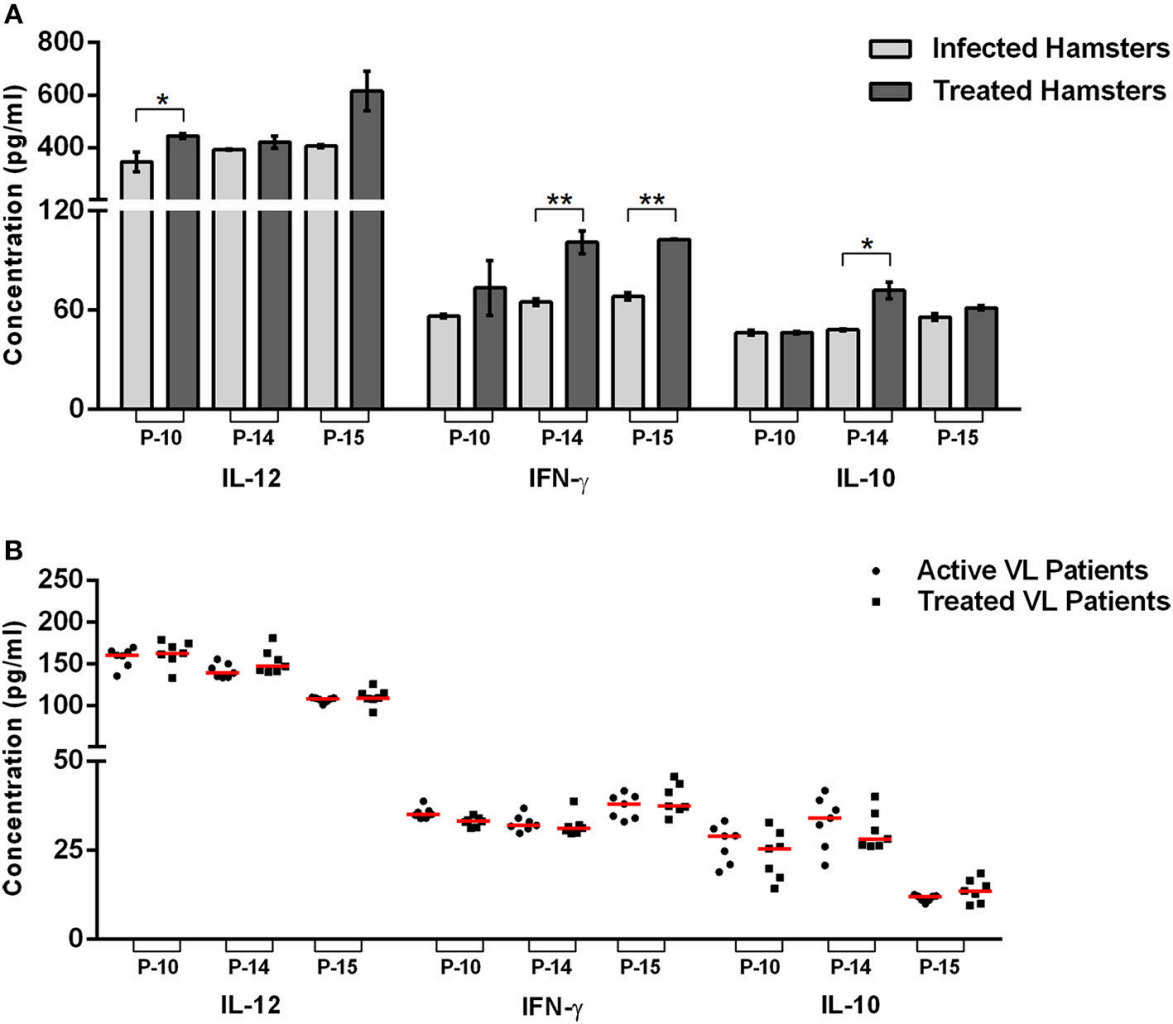
Cytokines level in the peptide-stimulated culture supernatant of lymphocyte derived from uninfected, infected, and treated *Leishmania*-infected hamsters **(A)** as well as in PBMCs derived from healthy contacts, active VL as well as treated VL patients **(B)**. The levels of significance were calculated using unpaired *t*-test between infected and treated groups (*p < 0.05 and **p < 0.01).

**Figure 3:**
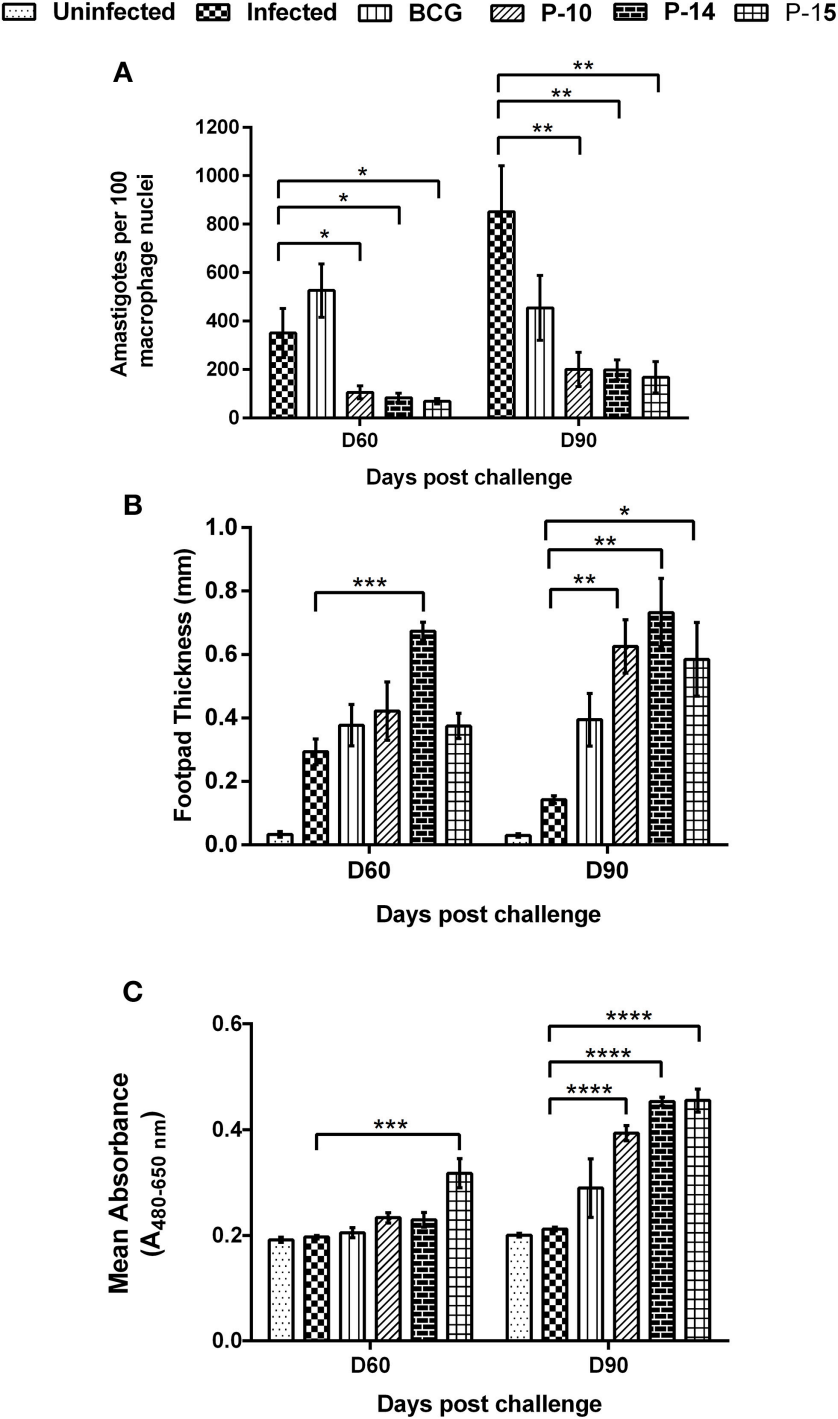
Parasite load in splenic dab smear of vaccinated hamsters **(A)**, DTH response to SLD as footpad swelling **(B)** and lymphoproliferative response in vaccinated hamsters against SLD **(C)** in comparison to the unimmunized infected hamsters on days 60 and 90 p.c. One-way ANOVA was used to calculate the statistical significance between the vaccinated and infected group (*p < 0.05; **p < 0.01; ***p < 0.001, and ****p < 0.0001).

**Figure 4:**
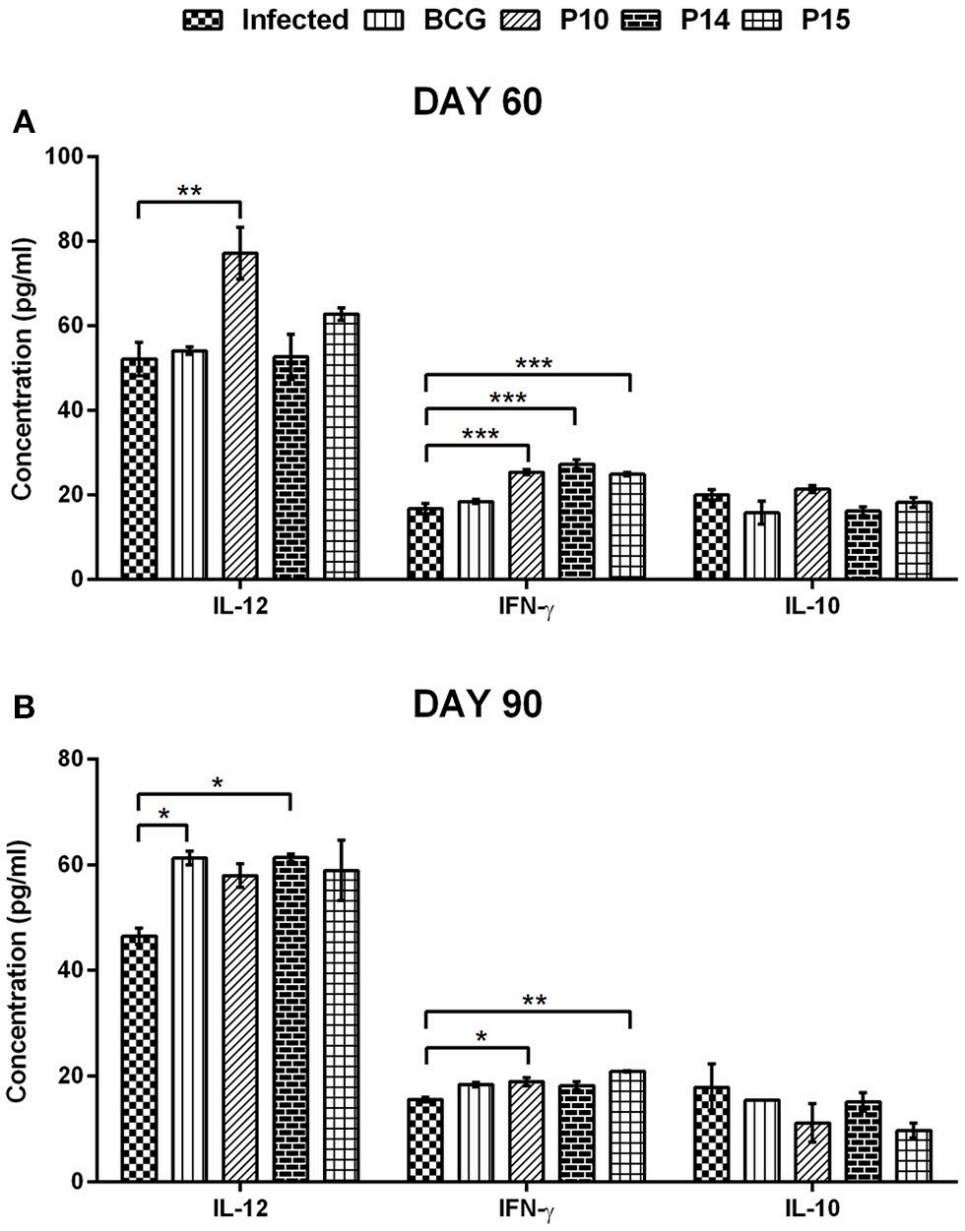
Cytokines level in serum samples of vaccinated hamsters at day 60 **(A)** and day 90 **(B)** p.c. Significance values indicate the difference in cytokine concentration between infected and treated hamsters (*p < 0.05; **p < 0.01; and ***p < 0.001) and was calculated using One-way ANOVA.

**Figure 5:**
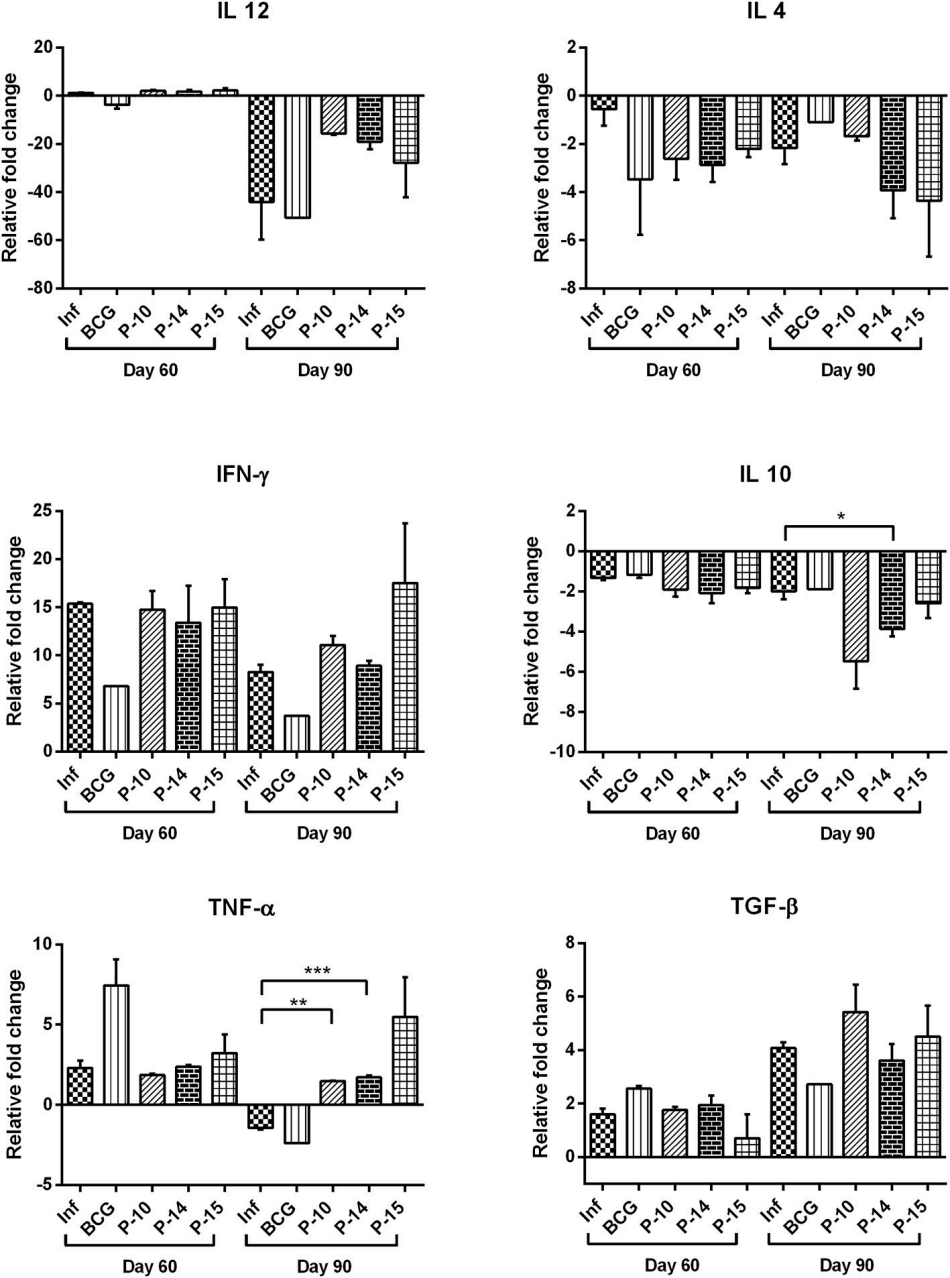
Cytokine mRNA expression profile of spleen of normal, infected, and vaccinated hamsters on days 60 and 90 p.c. by quantitative real-time-PCR. The levels of significance were calculated using unpaired *t*-test between infected and treated groups (*p < 0.05; **p < 0.01; and ***p < 0.001).

All the experiments were performed in two replicates. Statistical analysis was performed using GraphPad Prism 6.01 (GraphPad Software, San Diego, CA, USA). Results were expressed as mean ± SEM. Paired *t*-test was used to calculate statistical significant differences between mean values of treated groups stimulated with synthetic peptides and their respective recombinant proteins in Figure 1. Unpaired *t*-test was used to calculate the levels of significance between infected and treated groups in Figures 2, **5**. In case of Figures 3, 4, one-way ANOVA was used to calculate the statistical significance between the vaccinated and infected group. A *p* value of < 0.05 was considered significant.

## Results

### *In silico* Prediction of Promiscuous T-Cell Epitopes

IEDB and SYFPEITHI servers were used to predict immunogenic peptides from the full-length protein sequences of six potential Th1 stimulatory proteins (LdAld, LdEno, LdTPI, LdPDI, LdelF2, and Ldp45). Both servers yielded several peptides (>100) out of which six top scoring peptides were selected against the HLA alleles which occurs most frequently in global population as well as against those recently reported to be involved in susceptibility to *Leishmania* infection in Indian population (27). Also, top scoring peptides were subjected to BlastP search for the identification of identical or highly similar sequences into the human genome. Peptides exhibiting higher identity with human proteins (>85%) in BLAST search were not considered. Finally, a total of 36 15-mer MHC-II binding peptides (six peptides of each protein) were chosen for the experimental evaluation as listed in Table 2.

**Table 2:** Notation and sequence of peptides predicted against the alleles reported to be involved in susceptibility to *Leishmania* infection in Indian population.

**Proteins**	**S. No**.	**AA seq**	**Peptides**	**Proteins**	**S. No**.	**AA seq**	**Peptides**
Aldo	P-01	179–193	TLARYAILSQISGLV	PDI	P-19	85–99	KYQIKGFPTLYIFRN
	P-02	180–194	LARYAILSQISGLVP		P-20	311–325	HHVMETYTPVTAESV
	P-03	137–151	LDGYVKRASAYYKKG		P-21	212–236	AESVKRFLATAVLDY
	P-04	1–15	MSRVTIFQSQLPACN		P-22	174–188	NFVFVTDAAISPNDA
	P-05	261–275	YTVMTLARTMPAMLP		P-23	166–180	ADSLRTQMNFVFVTD
	P-06	263–277	VMTLARTMPAMLPGV		P-24	371–385	QNVMLLFYAPWCGHC
Eno	P-07	1–15	MPIRKVYAREVLDSR	elF-2	P-25	15–29	AFSLTRFANMYAAKF
	P-08	160–174	VLPFQEFMIAPTKAT		P-26	566–580	IYNVRAYLPVAESFG
	P-09	188–202	HALKVIIKSKYGQDA		P-27	189–203	RGRFFAFGRIFSGKV
	P-10	128–142	LYRYIAGLAGTKDIR		P-28	2–16	KGTVAIGSGLQAWAF
	P-11	184–198	SEVYHALKVIIKSKY		P-29	75–89	DPIYQIFDAVMNEKK
	P-12	336–350	ACNSLLLKINQIGTI		P-30	117–131	KTVMMKFLPAAETLL
TPI	P-13	204–218	AARLRILYGGSVSAG	p45	P-31	107–221	GIHVDGYCAVVAHTI
	P-14	201–215	ADVAARLRILYGGSV		P-32	150–164	LRQMRPGATIYQVTD
	P-15	45–59	TFVHIPLVQAKLRNP		P-33	172–186	HYKVTPVDGVLSHMM
	P-16	138–152	NQTAKVVLSQTSAIA		P-34	306–320	GEVVAHFKITVLISN
	P-17	22–36	IEKLVQVLNEHNISH		P-35	320–334	NKKIEPITGLKPQKA
	P-18	43–57	APTFVHIPLVQAKLR		P-36	214–228	KAQVWTLDIVMTSGK

### *In vitro* Evaluation of Peptides

The cellular response generated by mesenteric lymph node-derived mononuclear cells isolated from treated *Leishmania*-infected hamsters as well as PBMCs of treated *Leishmania* patients against thirty-six peptides was compared with their respective proteins by XTT, as shown in Figures 1A,B. In the case of hamsters, five peptides *viz*. P-10 of Enolase, P-14, P-15, and P-18 of TPI and P-19 of PDI induced significant cellular proliferation (mean OD ± SE 0.2199 ± 0.008, 0.2127 ± 0.006, 0.2143 ± 0.008, 0.2221 ± 0.009, and 0.2275 ± 0.009, respectively). However, in human PBMCs, ten peptides namely P-08, P-10, and P-11 of Enolase, P-14 and P-15 of TPI, P-26 and P-27 of elF-2, P-32, P-33, and P-34 of p45 induced significant lymphoproliferative response (0.3127 ± 0.053, 0.2831 ± 0.054, 0.2654 ± 0.047, 0.3301 ± 0.053, 0.3035 ± 0.056, 0.3075 ± 0.056, 0.2848 ± 0.052, 0.3587 ± 0.068, 0.3452 ± 0.066, and 0.2959 ± 0.050). Thus, among all the 5 and 10 peptides that were identified eliciting optimum cellular responses in treated groups of hamster and human, respectively, three peptides i.e., P-10, P-14, and P-15 were observed to be common in both. A significant proliferation was observed in patient's PBMCs as well as in hamster's mononuclear cells stimulated by standard mitogens (data not shown) indicating the experimental sensitivity.

Furthermore, cytokine production was estimated in cell culture supernatants of P-10, P-14, and P-15 stimulated cells. A significant production of Th1 cytokines (IL-12 and IFN-γ) in the supernatant of all the three peptides stimulated mono-nuclear cells derived from treated *Leishmania*-infected hamsters were noticed in comparison to the infected ones (Figure 2A). Amongst the three peptides, P-14, and P-15 induced significantly higher production of IFN-γ (101 ± 6.832 pg/mL and 102.8 ± 0.3368 pg/mL, respectively), in the treated group in comparison to infected control. However, in the case of P-10, there was an apparent increase in IFN-γ level but was not statistically significant. Likewise, the level of IL-12 was also found to be increased in treated hamsters stimulated with all the three peptides, wherein the values weresignificant in the case of P-10 (444.7 ± 9.533 pg/mL) only. Conversely, there was slight or no difference in the IL-10 level, a Th2 cytokine, between the infected and treated groups stimulated by P-10, P-14, and P-15. However, when assessed in PBMCs of treated VL patients, though, these peptides generated a slight increase in IL-12 level with decreased production of IL-10 (except P-15 stimulated cells), the values were statistically non-significant (Figure 2B). The level of IFN-γ was, however, found to be comparable to infected patients.

### *In vivo* Evaluation of Three Potential Peptides

Three potential peptides (P-10, P-14, and P-15), based on the results of *in vitro* study, were further subjected for their prophylactic efficacy alongwith BCG in Syrian golden hamsters.

#### Determination of Parasite Load, DTH, and Lymphoproliferative Responses

A significant inhibition of parasitic multiplication was noticed in the spleen of P-10+BCG and P-15+BCG (75% inhibition) and P-10+BCG (65% inhibition) vaccinated animals as compared to infected controls on day 60 p.c. (Figure 3A). By day 90 p.c., 75% parasite inhibition was noticed in all the three peptides+BCG vaccinated groups as compared to infected ones. In contrast, higher parasite load was observed in hamsters immunized with BCG alone in comparison to the three peptide+BCG vaccinated hamsters both on days 60 and 90 p.c.

DTH and *Leishmania*-specific lymphoproliferative responses, the indices of cell-mediated immunity, were measured in animals of each experimental group. Though, there was an apparent increase in DTH response in BCG and all the three peptides+BCG vaccinated animals in comparison to infected control on day 60 p.c.; it was significant in hamsters immunized with P-14+BCG only (Figure 3B). By the day 90 p.c., however, the response became significant in all the three peptides+BCG vaccinated groups (not in BCG alone group) when compared with infected controls. Nevertheless, a substantial increase in lymphoproliferative response, in comparison to infected control, was evident only in the lymph node cells of hamsters vaccinated with P-15+BCG (0.3175 ± 0.027) on day 60 p.c., whereas by day 90 p.c., this response was found to be highly significant in all the three peptides+BCG vaccinated groups i.e., P-10+BCG (0.3931 ± 0.014), P-14+BCG (0.4533 ± 0.008), and P-15+BCG (0.4551 ± 0.022) which was in accordance with the DTH response (Figure 3C). Lesser non-significant proliferation was observed in animals vaccinated with BCG alone (0.2048 ± 0.010 and 0.2895 ± 0.056 on days 60 and 90 p.c., respectively).

#### Measurement of Cytokine Response in Serum Samples of Vaccinated Hamsters

The skewness in the cytokine *milieu*, if any, was assessed in the serum samples of hamsters following immunization and challenge. There was significant production of IFN-γ in all the three peptides+BCG vaccinated groups—P-10+BCG (25.32 ± 0.608 pg/mL), P-14+BCG (27.26 ± 0.992 pg/mL), and P-15+BCG (24.88 ± 0.431 pg/mL) on day 60 p.c. in comparison to unvaccinated infected control group (16.73 ± 1.238 pg/mL) (Figure 4A). However, on day 90 p.c. the IFN-γ level was almost similar and observed to be significantly higher in P-10+BCG (18.96 ± 0.740 pg/mL) and P-15+BCG (20.91 ± 0.153 pg/mL) vaccinated groups only (Figure 4B). The IFN-γ level in only BCG vaccinated animals remains almost similar to infected control at both the observation points. In addition, P-10+BCG vaccinated hamsters also significantly induced IL-12 (77.12 ± 6.117 pg/mL) with a perceptible increase in P-15+BCG (62.73 ± 1.473 pg/mL) vaccinated group on day 60 p.c. whereas, its level was noticed to be significant in BCG alone (6.26 ± 1.26 pg/mL) and P-14+BCG (61.33 ± 0.700 pg/mL) vaccinated hamsters on day 90 p.c. as compared to unvaccinated infected control group. Conversely, the level of IL-10, a Th2 cytokine, in BCG alone and all the peptide+BCG vaccinated groups was more or less comparable to the infected control groups on both days 60 and 90 p.c.

#### Analysis of Cytokine Profiles in the Splenic Tissue of Vaccinated Hamsters

Cytokine production in all the experimental groups was further assessed at transcript levels by quantifying their expression levels in splenic tissue by qRT-PCR using RPL18 as an internal reference gene. Amongst Th1 cytokines, the mRNA expression of IFN-γ was observed to be similar in all the three peptides+BCG vaccinated groups as compared to that of infected control on day 60 p.c. which increased progressively by day 90 p.c especially in P-15+BCG vaccinated group (almost 2 fold). However, there was decreased mRNA expression of IFN-γ in BCG alone vaccinated group as compared to infected control at both observation points. Similarly, increased mRNA expression of IL-12 was noticed in all the three peptides+BCG vaccinated groups on day 60 p.c. but surprisingly by day 90 p.c. its expression was down-regulated in all the vaccinated groups. The expression of TNF-α, however, was found to be increased in all the three peptides+BCG vaccinated hamsters on day 90 p.c. which was significant only in P-10+BCG and P-14+BCG vaccinated groups in comparison to infected control group. On the contrary, the expression of Th2 cytokines, IL-4 and IL-10, in general, was down-regulated in all the vaccinated groups on both day 60 and 90 p.c., as compared to infected control whereas a slight increase in mRNA expression of TGF-β was observed in P-10+BCG and P-15+BCG by day 90 p.c (Figure 5).

## Discussion

Peptide-based synthetic vaccines offer a safer alternative to traditional vaccines as it utilizes shorter peptide fragments for eliciting an enhanced targeted immune response thus avoiding allergenic responses (28). The identification and proper selection of peptide antigens are, however, crucial for the development of an effective peptide-based synthetic vaccine (29). A large number of such vaccines have recently reached clinical trials (30). In case of leishmaniasis, several proteins such as Gp63, KMP-11, A2-protein, LPG, cysteine protease, etc. were explored for determining the potential T-cell activating peptides as reviewed by Joshi et al. and De Brito et al. (31, 32). Since the generation of Th1 biased cellular immunity is critical for mediating the protective response against intracellular pathogens including *Leishmania* (14), Th1 stimulatory peptides were, therefore considered to be suitable vaccine candidates. In our earlier study, we have identified six Th1 stimulatory proteins, which were found to elicit optimum cellular responses (17). Herein, we have further subjected these six proteins to bioinformatics analysis for the identification of potential T-cell epitopes.

Due to the existence of immense diversity among human leukocyte antigen (HLA) genes, they offer a selective advantage to the immune system against diverse microbial antigens (33). There are enormous evidence indicating a genetic association between HLA and numerous infectious diseases (34). Thus, knowledge of epitope recognition among the targeted human population is a critical factor in the designing of such vaccines (35). Thirty-six MHC II binding peptides were selected using IEDB and SYFPEITHI, keeping in view the fact that HLA-DRB1-HLA-DQA1 HLA class II region contributes to VL susceptibility in India (27). Also, MHC-II loaded antigen triggers CD4+ T-helper cells which further activate cellular as well as humoral immunity (36). Out of thirty-six peptides, three *viz* P-10, P-14, and P-15 were found to elicit a significant lymphoproliferative response in treated *Leishmania* exposed hamsters as well as human subjects as compared to their respective proteins. Although lymphoproliferative assay is a measure of T-cell function, heterogeneity of CD4+ T-cells modulates cytokine response either into protective or deleterious one (37) and therefore it was measured. A Th1 biased cytokine (IL-12 and IFN-γ) responses were observed in lymph node-derived mono-nuclear cells supernatant, incubated with these three peptides, from treated *Leishmania*-infected hamsters in comparison to infected one. However, in the case of PBMCs of treated VL patients, all the three peptides resulted in the increased generation of IL-12 with decrease production of IL-10 as compared to active VL patients, though statistically non-significant. The IFN-γ level was, however, found to be comparable with that of infected patients. A similar observation was made by Singh et al. (38) wherein no difference was observed in the IFN-γ level in SLA stimulated whole-blood cellsbetween active and cured VL patients in Indian Subcontinent although it was higher as compared to healthy subjects.

Encouraged with these observations, the prophylactic efficacies of these peptides were further evaluated in hamsters against *Leishmania* challenge. It is well-known that peptides are often weakly immunogenic when applied alone and thus require particulate carriers or adjuvants for enhancing their efficacy (29). In this study, BCG-an immunostimulatory adjuvant has been employed since it has been reported to be associated with induction of Th1 immune response *via* augmenting cell-mediated immune (CMI) response to the associated antigens (39) and is the most acceptable Th1 inducing adjuvant presently available for human use (40). The peptide vaccinated hamsters, in combination with BCG, offered a considerable protective response with 75% reduction in splenic parasitic load by day 90 p.c.

Since, chronic infections are usually characterized by T-cell exhaustion including loss of cellular proliferation and increase in pathogen burden, the development of strong CMI responses is believed to be contributing to healing *Leishmania* infection. In this study, the findings revealed that all the groups of peptide+BCG vaccinated hamsters have exhibited a significant lymphoproliferative response by day 90 p.c. which on the contrary, was severely impeded in the infected hamsters. Moreover, since, it has been postulated that the Th1 cell is the “inducer” of a DTH response (41) therefore, the hamsters of all the experimental groups were subjected to this hypersensitivity test. It was noticed that all the vaccinated hamsters elicited stronger DTH reaction as compared to infected control animals which showed the low level of parasite-specific DTH responses and this can be correlated with the disease progression.

Th1-type immunity, characterized by the production of IL-12 and IFN-γ, helps in the development of protective response against *Leishmania* infection (42). However, IL-10, an immunosuppressive cytokine, reduces the production of Th1 cytokines and their neutralization augments IFN-γ production and promotes amastigotes clearance (42). In our study, IFN-γ was found to be elevated in the splenic tissue as well as in the serum of all the peptide+BCG vaccinated groups by day 90 p.c. However, at the transcript level, IFN-γ was also found to be elevated in infected animals which were in accordance with the observation made by Melby et al. (43). IL-12, another Th1 cytokine, was found to be elevated in serum samples of vaccinated hamsters at both time points but at the transcript level, though it was initially found to be up-regulated on day 60 p.c., it eventually decreased by day 90 p.c. Amongst Th2 cytokines, IL-10 was found to be more or less similar in the serum samples of vaccinated and infected animals at both time points of observation. However, it was found to be down-regulated, at the transcript level, on day 60 and 90 p.c. which was in accordance to the observation made in treated VL patients (44). These differences in mRNA transcripts and serum cytokine levels might be due to various post-transcriptional, translational, and protein degradation regulatory mechanisms (45).

Furthermore, TNF-α reported to be associated with control of *Leishmania* infection through macrophage activation and tissue granuloma formation (46), was observed to be up-regulated in the splenic tissue of all the vaccinated hamsters indicating toward the generation of the Th1 type of immune response. On the other hand, among the Th2 cytokines, IL-4 was found to be down-regulated, at the transcript level, on day 60 and 90 p.c. However, the expression of TGF-β, which has been implicated in the susceptibility to VL due to its suppressor effects on macrophages during VL infection (47), on the contrary, was found to be almost similar or increased in peptide+BCG vaccinated groups at day 60 (P-10 and P-14) and day 90 (P-10 and P-15) as compared to infected control. This observation was in accordance with the findings reported by Araujo-Santos et al. (48).

Thus, the present study demonstrates that these three peptides (P-10, P-14, and P-15) with BCG offer noticeable immunoprotective responses in hamsters against experimental *Leishmania* challenge and thereby indicate toward their being the potential vaccine candidates. It is well-known that pathogen displays numerous copies of multiple antigens to the immune system and thus generate a holistic immune response. Therefore, it would be advisable to emphasize on the inclusion of multiple epitopes into a vaccine as it allows better coverage of antigen diversity of the pathogen and genetic polymorphism of the mammalian immune system. Keeping this in view, it would be worthwhile to develop a multi peptide-based synthetic vaccine conjugated with an immunostimulatory peptide so as to augment the protective immune response, thereby fortifying the host immunity in an effective way.

## Ethics Statement

All the animal-based experimental procedures using Syrian golden hamsters (*Mesocricetus auratus*, 6–8-weeks-old), including their numbers, were approved by institutional animal ethical committee (IAEC, Approval No IAEC/2013/79/dated 31/07/2013) regulating according to the guidelines of the committee for the purpose of control and supervision of experiments on animals (CPCSEA). Similarly, the protocols and study on human subjects, belonging to hyper-endemic areas of Bihar, were approved by Human Ethics Committee of the Kala-Azar Medical Research Center (KMRC), Muzaffarpur (Approval No EC-KAMRC/Vaccine/VL/2007-1). A written informed consent was taken from all the human subjects before their enrolment in this study.

## Author Contributions

SJ, NY, and KR performed the human and animal experiments. VK, MS, and SJ conducted the *in-silico* studies. RA synthesized peptides under the supervision of WH. SS provides human samples. SJ and AD analyzed the data and wrote the manuscript. AD, AS, and SJ designed the study. AD supervised the entire project.

### Conflict of Interest Statement

The authors declare that the research was conducted in the absence of any commercial or financial relationships that could be construed as a potential conflict of interest.
