# Supplementary material for: Immunogenicity and Protective Efficacy of T-Cell Epitopes Derived From Potential Th1 Stimulatory Proteins of Leishmania (Leishmania) donovani
Source: Front Immunol. 2019 Feb 28;10:288. doi: 10.3389/fimmu.2019.00288 (PMC6403406; doi:10.3389/fimmu.2019.00288)
Supplement: Supplementary file 1 [file Table_1.doc]

**Supplementary Table I:** Clinical information of infected and treated VL patients and endemic healthy contacts.

| **PATIENTS** | **AGE(yr)/GENDER** | **GROUP** | **DURATION OF TREATMENT WITH AMBISOME**  **(in months)** |
| --- | --- | --- | --- |
| **1** | 22/M | Infected | - |
| **2** | 10/M | Infected | - |
| **3** | 08/M | Infected | - |
| **4** | 11/M | Infected | - |
| **5** | 20/M | Infected | - |
| **6** | 32/F | Infected | - |
| **7** | 32/M | Infected | - |
| **8** | 10/M | Treated | 6 |
| **9** | 50/M | Endemic Contact | - |
| **10** | 50/M | Treated | 6 |
| **11** | 80/M | Treated | 6 |
| **12** | 15/M | Treated | 6 |
| **13** | 19/M | Endemic Contact | - |
| **14** | 12/F | Treated | 6 |
| **15** | 24/M | Endemic Contact | - |
| **16** | 18/M | Treated | 6 |
| **17** | 19/F | Treated | 6 |
| **18** | 40/M | Endemic Contact | - |
| **19** | 35/M | Endemic Contact | - |
| **20** | 47/M | Endemic Contact | - |
